# The Impact of Health Education on Hemoglobin A1C in Diabetic Patients at the Family Medicine Department of King Fahad Armed Forces Hospital in Jeddah

**DOI:** 10.7759/cureus.75627

**Published:** 2024-12-13

**Authors:** Khalid A Almotairy, Taroub T Sabbagh, Mashael A Alkhuli, Mie A Tallab, Ruba A Hawsawi, Noura A Baroom

**Affiliations:** 1 Family Medicine Department, King Fahad Armed Forces Hospital, Jeddah, SAU; 2 Health Education Department, King Fahad Armed Forces Hospital, Jeddah, SAU

**Keywords:** diabetes, hba1c, health education, quality of life, self-management

## Abstract

Background: Diabetes mellitus (DM) is a long-term condition associated with severe complications. Individuals with diabetes must make numerous self-management decisions and participate in diverse care activities. Diabetes self-management education and support assist patients in making these decisions and performing these activities, enhancing their health outcomes. The study aims to assess the effects of health education on median hemoglobin A1c (HbA1C) levels, the development of diabetic complications, and the number of hospital admissions in patients with uncontrolled type 2 DM.

Methods: This prospective quasi-experimental study, conducted at King Fahad Armed Forces Hospital in Jeddah, Saudi Arabia, from September 2020 to September 2022, assessed the impact of a structured diabetic education program on uncontrolled type 2 DM patients. The study involved 100 patients with HbA1c >8%: 50 in the intervention group who received the program and 50 in the control group who did not. HbA1c levels were measured before and after the intervention. Data was collected securely, and an experienced biostatistician performed statistical analysis.

Findings: The two groups found no significant differences in age, disease duration, HbA1c, medication type, insulin type, education level, employment, and clinical visits. However, the control group had significantly more females (40 (81.6%) vs. 32 (64%), p=0.049), and the intervention group had larger family sizes (43 (86%) with >4 members vs. 21 (42.9%), p<0.0001). The intervention group showed a significant decrease in HbA1c from baseline across all measurements post-educational program (p<0.0001), whereas the control group did not show significant changes. Economic status also differed significantly (p=0.024). No significant differences were found between groups in follow-up HbA1c measurements.

Conclusion: The study demonstrates that the diabetic education program at King Fahad Armed Forces Hospital effectively lowered HbA1c levels in type 2 DM patients, confirming the program's role in enhancing glycemic control through structured self-management education and support.

## Introduction

Diabetes mellitus (DM) is a chronic metabolic disorder resulting from complicated interactions of genetic, immunological, and environmental factors, leading to elevated blood glucose levels due to insufficient insulin production, resistance to insulin action, or both [1]. According to the International Diabetes Federation, diabetes affects approximately 9% of adults aged 20 to 79, with about 50% of cases going undiagnosed [2].

The prevalence of impaired glucose tolerance is expected to rise from 344 million in 2010 to 472 million by 2030, driven by increasing risk factors like obesity [3]. Type 2 DM, the most prevalent type, constitutes 95% of global diabetes cases [4]. In Saudi Arabia, a study published in 2011 found that the prevalence of type 2 DM was 30% among males and 27.6% among females [5]. Additionally, a community-based study conducted from 1995 to 2000 reported an overall prevalence of type 2 DM at 23.7% in Saudi Arabia, with rates of 26.2% in males and 21.5% in females [6].

Diabetes remains a significant global health challenge and ranks among the leading causes of global mortality. It affects millions worldwide and poses substantial economic and healthcare burdens. Type 2 DM, in particular, accounts for most diabetes cases globally and is associated with numerous complications if not adequately managed [7,8]. Diabetes can adversely affect both the overall health and the quality of life (QoL) of patients, particularly through long-term complications such as microvascular (e.g., retinopathy, nephropathy, and neuropathy) and macrovascular issues (e.g., myocardial infarction, angina pectoris, stroke). These complications significantly diminish patients' health-related QoL (HRQoL) [3]. Maintaining target hemoglobin A1c (HbA1c) levels is crucial for reducing such complications in diabetic patients [9].

For effective diabetes management, patients must understand the importance of diet, medication adherence, and adjustments according to their exercise regimen [10]. Globally, 34.3% of diabetes patients have limited health education [11]. Diabetes self-care education is crucial in achieving optimal control [12], with studies showing positive impacts on HRQoL [13]. However, less than half of diabetic patients acquire adequate knowledge and skills for diabetes control, and fewer than 50% achieve optimal glycemic control (HbA1c <7.0%) [14].

Diabetes educational programs have been recommended to enhance patients' HRQoL [15]. Increasing evidence suggests that programs addressing self-care and psychological support improve QoL and diabetes management [16]. Cultural contexts and healthcare systems can shape interactions between patients and healthcare providers, which can affect health literacy's impact on diabetic patients' health outcomes [4]. Despite Saudi Arabia's high diabetes incidence, few studies have explored health literacy's effects on HRQoL, self-care management, and glycemic control [17,18].

This study aimed to evaluate the impact of health education on various health outcomes among the target study group. The primary objective is to measure the effect of health education on the median HbA1C within the study period. The secondary objectives are to assess the impact of health education on diabetic complications and the number of hospital admissions within the same period.

## Materials and methods

Study design and setting

This prospective quasi-experimental study was conducted at the Family Medicine Department of King Fahad Armed Forces Hospital in Jeddah, Saudi Arabia, over two years from September 2020 to September 2022. The study involved 100 patients with uncontrolled type 2 DM: 50 in the intervention group who received the structured diabetic education and 50 in the control group who did not.

Ethical consideration

The study was approved by the Research Ethics Committee of the Medical Services Department for Armed Forces (approval number: REC620). Patient confidentiality was ensured by using anonymized patient record numbers. Informed consent was obtained from all participants or their legal guardians before their inclusion in the study.

Inclusion and exclusion criteria

The inclusion criteria for this study targeted patients with uncontrolled type 2 DM with an HbA1c level greater than 8% who were using oral medications and/or insulin. Patients were selected from the Chronic Disease Clinic Database at King Fahad Armed Forces Hospital using simple random sampling with a computer-generated random number list. Exclusion criteria ruled out patients with severe comorbid conditions such as advanced cardiovascular disease, including severe heart failure, recent myocardial infarction (within the past six months), severe valvular heart disease, and uncontrolled arrhythmias (e.g., recurrent atrial fibrillation with rapid ventricular response). Other excluded conditions included severe renal or liver disease, recent participation in other diabetic education programs, inability to adhere to the follow-up schedule, pregnant women, and patients under 18 or over 75 years of age.

Data collection

Data collection included demographic information, clinical parameters (HbA1c, blood pressure, body mass index), and diabetes management practices, with follow-up assessments at baseline, 3, 6, 9, 12, and 15 months to evaluate the intervention's effectiveness.

Diabetes education program

The structured diabetes education program was conducted over a two-year period, with sessions held once every month by two certified diabetes educators from the research team. The program content covered essential aspects of diabetes management, including guidance on lifestyle adjustments for diabetes control, target blood sugar ranges, and self-monitoring techniques. Instructions on recognizing and managing symptoms of hypo- and hyperglycemia were included. Patients were trained on using diabetes medications, including glucometers and insulin pens, with detailed demonstrations on injection sites, rotation techniques, and insulin dose titration to reach target blood glucose levels. Additional education covered foot care to reduce complications, strategies for HbA1c control, and the importance of annual check-ups. The benefits of lifestyle modifications in maintaining target blood sugar levels were also emphasized.

The sessions were delivered either in person at the diabetes education clinic or virtually by phone. Various teaching methods were employed for in-person sessions, including writing boards, photographs, videos, and printed handouts, all available in Arabic for accessibility. Hands-on demonstrations in the clinic helped ensure an accurate understanding and application of the techniques. For phone sessions, patients received educational materials in advance, either as hard copies or digitally through WhatsApp, allowing them to follow along with the educator during the call.

Variables

Primary variables included HbA1c levels as measured before and after the educational intervention. Secondary variables included diabetic complications, hospital admissions, and baseline characteristics such as age, gender, disease duration, medication type, insulin type, education level, employment status, family size, economic status, and clinical visits.

Statistical analysis

Data analysis was performed using SPSS Statistics version 26 (IBM Corp. Released 2019. IBM SPSS Statistics for Windows, Version 26.0. Armonk, NY: IBM Corp). Descriptive statistics included mean and standard deviation for continuous variables and frequency and percentage for categorical variables. The Shapiro-Wilk test was used to test normality. Group comparisons were conducted using chi-square tests for categorical variables and repeated measures ANOVA for continuous variables. Statistical significance was set at p<0.05.

## Results

Regarding the baseline characteristics of the two groups, no significant differences were observed in age, duration of disease, HbA1c, medication type, type of insulin, education level, employment, and clinical visits. However, a statistically significant difference was noted between them in terms of gender, as 40 females (81.6%) were in the control group, compared to 32 females (64%) in the intervention group (p=0.049). A significant difference was also observed regarding family size, where the majority of the intervention group (43 participants, 86%) had more than four family members, while only 21 participants (42.9%) in the control group had the same family size (p<0.0001). Additionally, the economic status differed significantly between the groups, with 23 participants (46%) in the intervention group being classified as low economic status, compared to 13 (26.5%) in the control group (p=0.024). Moreover, there was a significant difference in complications reported before the intervention: 15 participants (30%) in the intervention group reported microvascular complications, compared to 28 controls (56%) (p=0.002). However, no significant difference was observed between both groups regarding the number of hospital admissions after the intervention (Table 1).

**Table 1:** Comparison between intervention and control groups regarding baseline characteristics ^a^: Microvascular complications included diabetic retinopathy, nephropathy, and neuropathy; ^b^: Macrovascular complications comprised cardiovascular disease (e.g., ischemic heart disease), cerebrovascular disease, and peripheral artery disease; * Significant p-value p<0.05

Variable	Intervention (n=50)		Control (n=50)		p-value
	Number	Percentage	Number	Percentage	
Gender					0.049*
Female	32	64	40	81.6	
Male	18	36	9	18.4	
Age (years)					1
<30	0	0	0	0	
30-40	0	0	0	0	
41-50	2	4.2	2	4.1	
51-60	22	44.8	23	46.9	
>60	25	51	24	49	
Duration of disease (years)					0.395
≤5	2	4	4	8.2	
6-10	12	24	6	12.2	
11-15	7	14	12	24.5	
16-20	11	22	9	18.4	
>20	18	36	18	36.7	
HbA1c level (%)					0.45
8-9%	10	20.4	8	16.3	
9.1-10%	8	16.3	11	22.4	
10.1-11%	10	20.4	15	30.6	
>11%	21	42.8	15	30.6	
Medication type					0.122
Oral	9	18	3	6.1	
Insulin	5	10	3	6.1	
Both	36	72	43	87.8	
Type of insulin					0.121
Long acting	15	36.5	11	24	
Intermediate	2	4.9	1	2.1	
Basal bolus	24	58.5	34	73.9	
Educational level					0.216
Illiterate	21	42	27	55.1	
Elementary school	14	28	7	14.3	
Junior high school	3	6	7	14.3	
High school	9	18	7	14.3	
University degree	3	6	1	2	
Employment status					0.14
Employed	2	4	3	6.1	
Unemployed	29	58	36	73.5	
Retired	19	38	10	20.4	
Family size					<0.0001*
Living alone	0	0	1	2	
2-4 members	7	14	27	55.1	
>4 members	43	86	21	42.9	
Economic status					0.024*
Low	23	46	13	26.5	
Medium	25	50	36	73.5	
High	2	4	0	0	
Clinical visits					0.254
Physician	16	32	21	43.8	
Dietician	0	0	0	0	
Both	34	68	27	56.2	
Complications before the intervention					0.002*
None	26	52	22	44	
Microvascular ^a^	15	30	28	56	
Macrovascular ^b^	6	12	0	0	
Both	3	6	0	0	
Number of hospital admissions after the intervention					0.6
0	49	98	48	96	
1	1	2	2	4	

**Figure 1:**
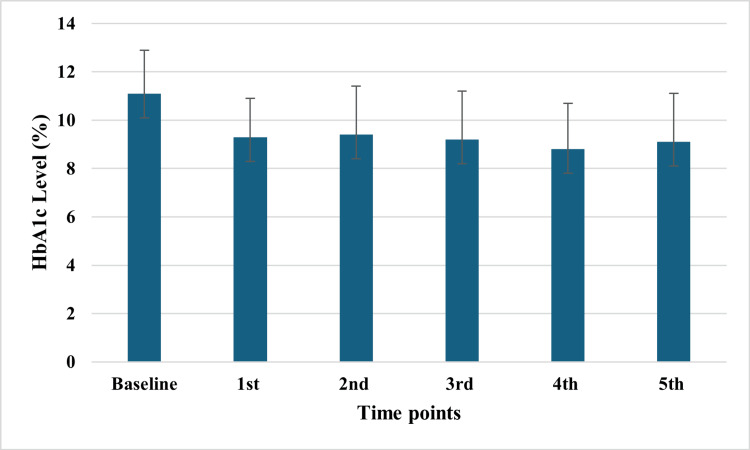
Decrease of HbA1c over time after application of educational program in the intervention group HbA1c: hemoglobin A1c

**Figure 2:**
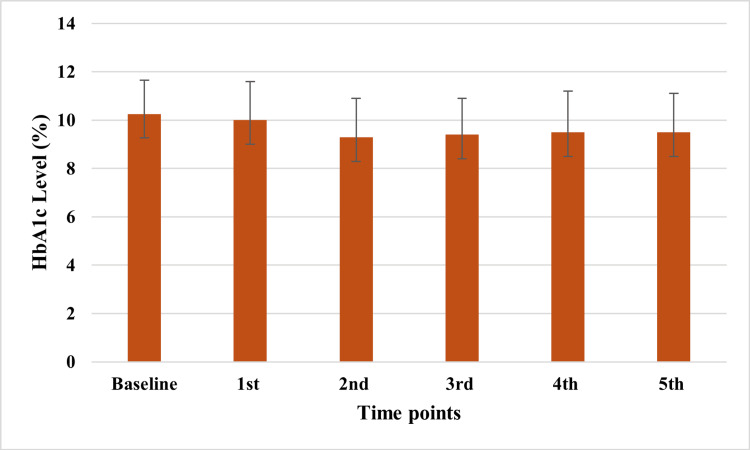
Different measurements of HbA1c in the control group HbA1c: hemoglobin A1c

After applying the educational program in the intervention group, there was a statistically significant decrease in HbA1c from baseline in all five measurements done thereafter (Figure 1 and Table 2). On the other hand, no statistically significant differences were observed in the HbA1c measurements done in the control group compared to the baseline (Figure 2 and Table 2).

**Table 2:** Comparison between intervention and control groups regarding change from baseline follow-up measurements of HbA1c HbA1c: hemoglobin A1c

Group	Baseline	1^st^	2^nd^	3^rd^	4^th^	5^th^
Intervention	11.1 ± 1.8	9.3 ± 1.6	9.4 ± 2	9.2 ± 2	8.8 ± 1.9	9.1 ± 2
Difference		-1.74 ± 0.28	-1.65 ± 0.35	-1.88 ± 0.31	-2.3 ± 0.33	-1.97 ± 0.37
p-value		<0.0001	<0.0001	<0.0001	<0.0001	<0.0001
Control	10.26 ± 1.4	10 ± 1.6	9.3 ± 1.6	9.4 ±1.5	9.5 ± 1.7	9.5 ± 1.6
Difference	-	-0.25 ± 0.28	-0.9 ± 0.29	-0.89 ± 0.3	-0.79 ± 0.38	-0.7 ± 0.36
p-value	-	1	0.06	0.13	0.7	0.9

**Figure 3:**
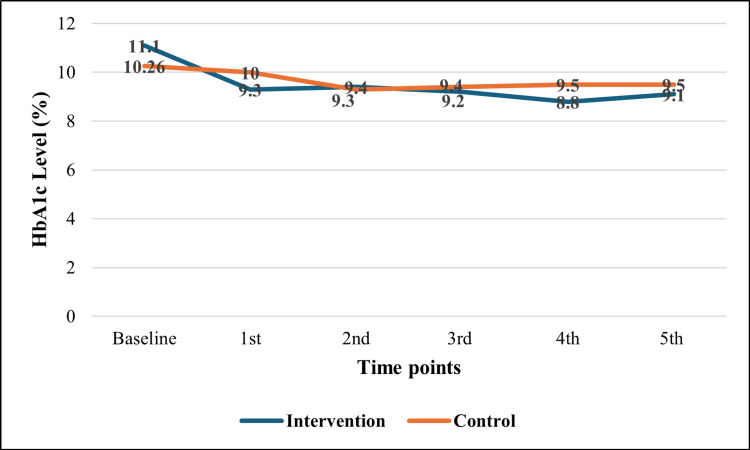
Comparison between intervention and control groups regarding follow-up measurements of HbA1c HbA1c: hemoglobin A1c

Figure 3 shows no significant difference between the two groups regarding different HbA1c measurements at different time points.

## Discussion

This study aimed to evaluate the effectiveness of a structured diabetic educational program in improving glycemic control, measured by HbA1c levels, and its impact on diabetic complications and hospital admissions.

The study results demonstrated a significant reduction in HbA1c levels in the intervention group following the structured educational program. Specifically, HbA1c levels decreased significantly from baseline across all five follow-up measurements (p<0.0001), indicating improved glycemic control post-intervention. These findings align with previous research showing a direct relationship between structured education and improved HbA1c levels [2,3,13]. Additionally, a recent Egyptian quasi-experimental study determined that interactive instructional interventions can effectively change behavior, lower HbA1c levels, and improve QoL for patients with diabetes [12]. An Iranian experimental study also reported that participants' knowledge, attitudes, and self-care practices improved significantly following structured self-care education, reducing average HbA1c levels [19]. Results from another present pretest experimental Saudi Arabian study concluded that diabetes education and patient-centered care significantly improved glycemic and cardiovascular risk reduction outcomes [20]. Adding more to this evidence, Shiferaw et al. declared in their meta-analysis that educational interventions can potentially enhance glycemic control levels in type 2 diabetes patients. Furthermore, the data indicated that educational interventions could improve disease knowledge among patients with type 2 DM [21]. Another meta-analysis done by Doshmangir et al. also suggested that theory- and model-based lifestyle therapies have been shown to improve HbA1c indices in patients with type 2 DM. Health education theories have promoted lifestyle change among patients with type 2 DM [22]. These studies emphasize that interactive educational interventions tailored to diabetes management can enhance patients' self-care behaviors, leading to better adherence to treatment regimens and lifestyle modifications.

In the current study, the educational sessions emphasized essential components of diabetes management: dietary modifications, physical activity, medication adherence, and self-glucose monitoring. These elements are critical in achieving better glycemic control and reducing diabetes-related complications. International guidelines recommend these components for improving glycemic control and reducing diabetes-related complications [23]. Findings of a recent meta-analysis highlighted that dietary education treatments spanning at least three months were highly effective in lowering HbA1c levels. Individualized education was more effective, and contact or non-contact education might be used to accomplish this [24].

In this study, the structured diabetes education program was administered to the intervention group. The structured educational program encompasses interactive workshops, personalized counseling sessions, and group discussions. Patients should be encouraged to set achievable dietary adjustments and physical activity goals, with regular follow-ups to track progress and offer assistance. The program shall also include training on properly utilizing glucose monitors, emphasizing the importance of consistent monitoring to track blood sugar levels.

Educating patients about medication adherence, including different types of diabetes medications, their mechanisms, and the significance of adhering to prescribed regimens, is another crucial aspect. This comprehensive approach, addressing various facets of diabetes management, may significantly contribute to the program's effectiveness. These educational programs may enhance glycemic control by equipping patients with essential tools and knowledge to make informed health decisions. This holistic strategy ensures patients are well-informed and empowered to manage their health, resulting in improved outcomes and a better QoL [2,3]. The study underscores the critical role of structured education in advancing diabetes care and emphasizes the value of a multi-dimensional approach to patient education.

In addition to evaluating glycemic control, this study observed a noticeable trend towards fewer admissions in the intervention group, suggesting potential benefits that require further investigation with larger sample sizes. However, no statistically significant differences in hospital admissions were observed between the intervention and control groups. However, a retrospective study from Healy et al. showed that after adjusting for other variables in the final models, individuals with poorly managed diabetes who received formal diabetes education had a 34% lower risk of all-cause readmissions by 30 days and a 20% lower risk of readmissions by 180 days. This implies that inpatient instruction provides long-term benefits; however, the 180-day model was borderline significant [25]. Rubin and Shah also reported that inpatient diabetes education, diabetes management services, transition of care support, and outpatient follow-up are all related to a lower likelihood of acute care re-utilization [26]. Since both of these studies have focused on hospital readmissions following diabetes education provided during inpatient care, there is a need for further research in outpatient settings. This additional research is necessary to thoroughly assess and understand the outcomes of diabetes education interventions when administered in outpatient departments.

Diabetic complications such as neuropathy, nephropathy, retinopathy, and cardiovascular diseases present substantial risks to individuals with diabetes. These conditions are frequently exacerbated by inadequate management of blood sugar levels, underscoring the critical importance of achieving and maintaining optimal glycemic control to mitigate these health risks effectively [3,9]. Although this study did not directly measure complications, the observed improvement in glycemic control is expected to mitigate these risks over the long term.

Health literacy, the ability to access, understand, and apply health information to make informed decisions, is crucial in promoting self-management behaviors and achieving optimal health outcomes in chronic diseases like diabetes [4,17]. The theoretical framework of health literacy, as proposed by Paasche-Orlow and Wolf, underscores that improving patients' knowledge and skills through education can empower them to effectively manage their condition [27]. The relationship between health literacy and self-care management skills is often debated. However, our study supports that tailored educational interventions can bridge knowledge gaps and significantly improve patients' abilities to manage diabetes effectively. By focusing on personalized education, we found that patients could better understand and implement diabetes management strategies, leading to improved health outcomes and greater self-efficacy in managing their condition [4].

The implications of our study extend to clinical practice and public health policy in Saudi Arabia and similar healthcare settings globally. Implementing structured diabetes education programs in outpatient settings, such as family medicine departments, can potentially reduce healthcare costs associated with diabetes management by preventing complications and reducing hospital admissions. Healthcare providers, including physicians, nurses, and nutritionists, are pivotal in educating patients about diabetes self-management strategies. Multidisciplinary approaches that involve collaboration between healthcare professionals and patients can optimize diabetes care delivery and improve patient outcomes [28-30].

Strengths and limitations

The study effectively highlights the impact of a structured diabetic education program on improving glycemic control among type 2 DM patients, as evidenced by significant reductions in HbA1c levels in the intervention group. The study's robust methodology, including a quasi-experimental design and the use of a control group, enhances the reliability of the findings. Additionally, the study’s focus on a specific population at King Fahad Armed Forces Hospital in Jeddah allows for targeted insights into the effectiveness of health education within a localized healthcare setting. The comprehensive approach to diabetes management, emphasizing dietary modifications, physical activity, medication adherence, and self-glucose monitoring, aligns with international guidelines and underscores the program's comprehensiveness.

Despite its strengths, the study has some limitations. The sample size of 100 patients, although balanced between intervention and control groups, may be insufficient to detect broader trends or smaller effect sizes, particularly regarding secondary outcomes like hospital admissions and diabetic complications. The study's retrospective design and potential selection biases, given the non-randomized nature of participant assignment, could affect the generalizability of the results. Additionally, significant differences in baseline characteristics, such as gender distribution and family size, between the groups might have influenced the outcomes.

Recommendations

Future studies should include larger and more diverse samples to enhance generalizability and employ randomized controlled trials to mitigate selection biases and establish causal inferences. Extending follow-up periods would allow assessment of long-term effects on glycemic control and complications. Broadening outcome measures to include QoL and patient satisfaction, utilizing multidisciplinary approaches, and addressing baseline differences with statistical techniques like propensity score matching is essential. Tailoring educational interventions to improve health literacy, as proposed by frameworks like Paasche-Orlow and Wolf, would enhance effectiveness. Policymakers should integrate structured diabetes education programs into standard care protocols to improve patient outcomes and reduce healthcare burdens.

## Conclusions

The study underscores the pivotal role of education in improving glycemic control and potentially mitigating long-term complications associated with diabetes. By integrating structured educational programs into clinical practice, healthcare providers can effectively empower patients with essential self-management skills. This approach not only enhances patients' ability to manage their condition but also contributes to better overall health outcomes. Educating patients about diet, medication adherence, and self-monitoring fosters proactive health management, reduces the risk of diabetes-related complications, and improves QoL.
